# Household and area income levels are associated with smoking status in the Korean adult population

**DOI:** 10.1186/s12889-015-1365-6

**Published:** 2015-01-31

**Authors:** Woo-Jun Yun, Jung-Ae Rhee, Sun A Kim, Sun-Seog Kweon, Young-Hoon Lee, So-Yeon Ryu, Soon-Woo Park, Dong Hyun Kim, Min-Ho Shin

**Affiliations:** Department of Preventive Medicine, Chonnam National University Medical School, Hak-1-dong, Dong-gu, Gwangju, 501-746 Republic of Korea; Jeonnam Regional Cancer Center, Chonnam National University Hwasun Hospital, Jeollanamdo, 519-809 Republic of Korea; Department of Preventive Medicine & Institute of Wonkwang Medical Science, Wonkwang University School of Medicine, Iksan, 570-711 Republic of Korea; Department of Preventive Medicine, Chosun University Medical School, Gwangju, 501-759 Republic of Korea; Department of Preventive Medicine, Catholic University of Daegu School of Medicine, Daegu, 705-718 Republic of Korea; Department of Social Medicine, Hallym University College of Medicine, Chuncheon, 200-702 Republic of Korea; Center for Creative Biomedical Scientists, Chonnam National University, Gwangju, 501-809 Republic of Korea

**Keywords:** Smoking, Household income, Area-level income, Multilevel analysis

## Abstract

**Background:**

Some previous studies have suggested that area-level characteristics have effects on smoking. The aim of this study was to evaluate the associations between household income and area income on smoking in Korean adults.

**Methods:**

This study was based on the Korean Community Health Survey (KCHS) performed in South Korea, between September and November 2009. In total, 222,242 subjects (103,124 men and 119,118 women) were included in the analysis. Information on smoking status was collected using a standardized questionnaire. Income status was determined by monthly household income. Household income was categorized as: <1 million won; <2 million won; <3 million won; and ≥3 million won. Area-level income categorized as quartiles. Data were analyzed using multilevel regression models. The analysis was conducted separately urban and rural, by sex.

**Results:**

The lowest household income group had a higher risk of smoking than the highest household income group in both urban and rural areas for both men and women after adjusting for individual characteristics (urban men: odds ration [OR], 1.44; 95% confidence interval [CI], 1.36–1.53; rural men: OR, 1.33; 95% CI, 1.25–1.42; urban women: OR, 2.38; 95% CI, 2.06–2.76; rural women: OR, 1.51; 95% CI, 1.25–1.83). In men, the lowest area-level income group had a higher risk for smoking than the highest area-level income group in urban areas after adjusting for individual characteristics and household income (OR, 1.17; 95% CI, 1.02–1.33). In women, the lowest area-level income group had a lower risk for smoking than the highest area-level income group in rural areas after adjusting for individual characteristics and household income (OR, 0.52; 95% CI, 0.39–0.70). However, no association was observed between area-level income and smoking in rural areas for men or in urban areas for women.

**Conclusions:**

The results showed that smoking is strongly associated with household income status in both men and women, and area-level income is partly associated with smoking. Effects of area-level income on smoking differed by sex and region. These findings suggest that area characteristics have contextual effects on health related behavior independent of individual characteristics.

## Background

Smoking is an important issue in public health. As it is a major risk factor for cardiovascular disease and is a common cause of death [1-3]. Annually, 10% of deaths in the world are was due to smoking, representing an estimated 6 million people [4].

Although the prevalence of smoking has decreased, the prevalence of smoking in Korea is still higher than in other OECD countries [5]. In many countries including Korea, the decline in smoking rate is greater in men than in women; indeed, the smoking rate in women in Korea has been increasing over the last 10 years [5]. According to the Korea National Health and Nutrition Examination Survey (KNHANES), the prevalence of smoking in men decreased from 66.3% to 45.7% during 1998–2007, whereas the prevalence of smoking in women increased from 6.5% to 7.4% during 1998–2008. However, the smoking rate in men has not decreased since 2008 [6].

Smoking is associated with many individual-level socioeconomic indicators such as income, education, and occupation, and it is more frequent among socioeconomically disadvantaged people [7-9]. Previous studies have shown that health behaviors are influenced by the social and structural environment in addition to individual-level characteristics, and some studies have reported that not only individual-level but also area-level socioeconomic characteristics have effects on smoking [10-12]. Understanding the contributions of individual or area indicators of socioeconomic status (SES) on smoking may help identify interventions and polices to reduce smoking.

Despite an increasing number of studies examining area effects on smoking, few studies have been conducted in Korea. Two previous studies evaluated area effects on smoking in Korea [13,14]. Kim et, al. evaluated the relationship between the residential distribution of those with high SES and smoking, but their study was restricted to one city and the area unit was a relatively small area compared to this study [13]. Park et, al. evaluated area deprivation, individual socioeconomic position and smoking, but all subjects in that study were women [14].

The important indicators of SES include education, occupation, and income, and these indicators are interrelated [15]. In many studies, education has been the most widely used indicator of the effects of SES on health behaviors [16]. However, other indicators of socioeconomic position may also provide additional information on the association between SES and health behaviors [17]. More clearly than other indicators of SES, income is associated with usable material and financial resources [18]. Income has effects on health behaviors such as smoking and alcohol intake because affects access to material resources related to health [19]. The lack of material resources may affect health behaviors through financial restrictions that prevent healthy choices [17].

The objective of this study was to investigate the cross-sectional association of individual-level and area-level income status with smoking after controlling for individual-level characteristics related to smoking in a national-representative sample of Korean adults.

## Methods

### Subjects

This study was based on a community health survey conducted in South Korea between September and November 2009 [20]. The KCHS is an annual nationwide survey conducted in every administrative districts since 2008 to provide to provide country-level health indicators of adults aged 19 years or older [20]. A complex, multistage probability sampling design was used to select participants. South Korea has 8 provinces, 1 special autonomous province, 6 metropolitan cities, and 1 special city, which are further divided into 253 administrative districts such as city, county and district. The KCHS conducted in every administrative district in South Korea. Overall completion rate of 2009 KCHS was 96.9% and rate of replaced of sampled household was 18.7% [20]. A total of 230,715 subjects participated in the 2009 KCHS. A total of 8,473 subjects were excluded from the study because of missing information on smoking and income status. Finally, the analysis included 222,242 subjects (103,124 men and 119,118 women). Urban and rural areas were classified at the sampling stage before participants were selected. Urban areas are cities and rural areas are a geographic area that is located outside cities. Definition of city is based on the population density of an area and city is defined as having over 100,000 population. No institutional review board approval was required for this study because the data are available for public use and are void of identifiers.

### Definition of smoking

Smoking was assessed using two questions: “Have you smoked more than 100 cigarettes in your lifetime?” and “Do you smoke currently?” Smoking was defined as smoking ≧100 cigarettes in a lifetime and currently smoking [21].

### Definition of income status

Income was measured by asking participants, “How much is your total household income per year (or per month), including income from all sources for all household members?” household income was determined by each subject’s self-reported monthly self reported household income in Korean won and was categorized as: <1 million won; <2 million won; <3 million won; and ≥3 million won.

Area-level income was determined by each area’s average household income calculated from the individual incomes of subjects who lived in the area, and was categorized as quartiles. First quartile is highest area-level income, and forth quartile is lowest one.

The correlation between individual income and area-level income is 0.952 (*p*=0.001).

### Covariates

Marital status was coded into two categories (married/partnered, never married/non-partnered). Family type was categorized as one generation, two generations, and three or more generations according to the number of generations of household members living together. Monthly drinking was defined as drinking alcohol at least once per month in the previous year. Monthly drinking (yes/no) was considered as drinking status in this study. Body mass index (BMI) was calculated using self-reported height and weight. Self-rated health status was measured from the question, “What do you think your health status is in your daily life?” Responses ranged along a 5-point scale from “very good” to “very bad” and were coded into two categories (1/2, 3–5). Stress perception status was measured from the question, “How much do you feel stress in your daily life?” Responses ranged from “none” to “serious” along a 4-point scale and were coded into two categories (1/2, 3/4). Perception of depressive mood was measured from the question, “Have you felt sadness or despair during two or more weeks in the last year?” Responses were coded into two categories (yes, no).

These definitions of smoking, income, and covariates were based on the Community Health Survey, 2009, Korea Centers for Disease Control and Prevention [22].

### Statistical analysis

All analyses in this study were performed separately for the two sexes and for urban and rural areas because of regional differences in smoking rate according to sex and socioeconomic status (SES). The reason why the analysis was conducted separately urban and rural was that urban areas have higher population density and vast human features, however, rural areas have a low population density and small settlements. There may be different social, economical, and cultural different behaviors between residents of urban areas and rural areas. The characteristics of the study population are expressed as mean ± standard deviation or number (weighted percentage). Age-adjusted prevalence rates for smoking according to income status were calculated using the standardized population data of Korea in 2005.

Multilevel logistic regression (random intercept) was used to evaluate the associations of household income and area-level income with smoking. Model 1 was fit with household income adjusted for individual-level characteristics such as age, family type, marital status alcohol intake, BMI, stress perception, depressive mood perception, and self-rated health status. Model 2 was fit with area-level income. Model 3 was fit with both household income and area-level income.

In addition, we evaluated the interaction of sex and urbanization with income variables on smoking. All statistical analyses were performed using STATA software, version 11.0 SE (STATA, College Station, TX, USA).

## Results

### Characteristics of the study population

Both men and women in rural areas were older than those in urban areas (men: 45.4 ± 15.1 vs. 52.8 ± 16.3 years; women: 46.3 ± 16.1 vs. 55.4 ± 17.1 years) (Table 1).

**Table 1:** **General characteristics of the study population (n=222,242)**

**Characteristics**	**Men**		**Women**	
	**Urban**	**Rural**	**Urban**	**Rural**
	**(n=57,411)**	**(n=45,713)**	**(n=65,664)**	**(n=53,454)**
Age (years)	45.4± 15.1	52.8 ± 16.3	46.3 ± 16.1	55.4 ± 17.1
Smoking status				
Never smokers	14,746 (27.2)	11,796 (26.4)	61,998 (94.6)	50,734 (94.8)
Former smokers	15,290 (24.7)	12,894 (24.8)	1,136 (1.7)	825 (1.6)
Current smokers	27,375 (48.1)	21,023 (48.8)	2,530 (3.7)	1,895 (3.6)
Household income (won)				
≧3 million	20,695 (38.7)	9,187 (24.8)	22,628 (37.4)	9,533 (22.3)
<3 million	14,364 (25.6)	8,781 (22.1)	15,416 (24.0)	9,029 (19.6)
<2 million	13,843 (23.4)	12,110 (27.6)	15,092 (22.6)	12,364 (24.7)
<1 million	8,509 (12.3)	15,635 (25.5)	12,528 (16.0)	22,528 (33.4)
Marital status				
Married/partnered	39,607 (65.3)	33,796 (67.8)	41,177 (62.1)	33,988 (63.8)
Never married/non-partnered	17,762 (34.7)	11,862 (32.2)	24,436 (37.9)	19,429 (36.2)
Family type				
One generation	14,713 (22.3)	20,111 (34,2)	16,481 (21.8)	24,669 (36.9)
Two generation	36,532 (66.8)	19,462 (51.4)	40,532 (65.1)	20,912 (47.3)
Over three generation	6,161 (10.9)	6,139 (14.4)	8,643 (13.1)	7,871 (15.8)
Monthly alcohol intake	42,390 (75.5)	29,500 (67.9)	25,371 (40.7)	14,570 (31.1)
Body mass index (kg/m^2^)	23.9 ± 5.7	25.2 ± 12.2	23.4 ± 9.3	28.3 ± 20.3
Stress perception	17,049 (30.6)	10,152 (25.4)	18,559 (28.8)	13,051 (26.2)
Depressive mood perception	3,202 (5.4)	2,267 (5.2)	6,493 (9.8)	4,760 (9.2)
Self-rated health status (average or good)	28,017 (51.1)	20,269 (48.6)	26,939 (43.4)	17,664 (37.0)

Data are presented as mean (standard deviation) or unweighted number (weighted percent).

Monthly drinking was defined as drinking alcohol at least once per month in the previous year. Self-rated health was categorized as average or good (very good, fairly good, and average) and poor (fairly poor and poor). Stress perception was measured with a 4-point scale and categorized as either high or low. Perception of depressive mood was categorized as either “yes” or “no”.

Both men and women in urban areas had higher household income than those in rural areas. (men: 38.7% vs. 24.8%; women: 37.4% vs. 22.3% for highest household income) (Table 1).

### Age-adjusted prevalence of smoking

Table 2 showed that the lowest household income group had higher age-adjusted prevalence of smoking than the highest household income group in both urban and rural areas for men and women.

**Table 2:** **Age-adjusted prevalence of current smoking**

**Characteristics**	**Men**		**Women**	
	**Urban**	**Rural**	**Urban**	**Rural**
	**(n=57,411)**	**(n=45,713)**	**(n=65,664)**	**(n=53,454)**
**Household income (won)**				
≧3 million	43.4	47.1	1.8	2.1
<3 million	48.1	51.4	3.3	2.7
<2 million	52.7	54.0	5.1	3.6
<1 million	56.0	53.8	8.6	5.9
**Area-level income (won)**				
1st quartile: 3.13to 5.58 million	46.5	50.7	3.6	4.4
2nd quartile: 2.69 to 3.12 million	49.2	52.4	3.9	3.9
3rd quartile: 2.21 to 2.68 million	49.9	52.5	3.7	3.1
4th quartile: 1.32 to 2.21 million	51.1	50.0	3.7	2.4

However, Table 2 showed that the lowest area-level income group had higher age-adjusted prevalence of smoking than the highest area-level income group only in urban area for men (first income quartile: 46.5%; fourth quartile: 51.1%), and, the highest area-level income group had higher age-adjusted prevalence of smoking than the lowest area-level income group in rural area for women (first income quartile: 4.4%; fourth quartile: 2.4%).

### Odds ratio for current smoking according to income status

There were interactions between household income and sex (*p*<0.001), and between household income and urbanization exists (*p*<0.001). However, there was no interaction between area-level income and sex (*p*=0.968) while there was interaction between area-level income and urbanization exists (*p*=0.039). So we analyzed the data separately for sex and urbanization.

In men, the lowest household income group had a higher risk of smoking than did the highest household income group in both urban and rural areas after adjusting for individual characteristics (urban: OR, 1.44; 95% CI, 1.36–1.53; rural: OR, 1.33; 95% CI = 1.25–1.42). The lowest area-level income group had a higher risk of smoking than did the highest area-level income group in urban, but not in rural areas after adjusting for individual characteristics and household income (urban: OR, 1.17; 95% CI, 1.02–1.33; rural: OR, 0.99; 95% CI, 0.89–1.10) (Table 3).

**Table 3:** **Odds ratio for current smoking according to income status in men**

	Model 1*	**Model 2**	**Model 3**
**Urban**			
**Household income**			
≧3 million won	1.00 (Reference)		1.00 (Reference)
<3 million won	1.23 (1.18-1.29)		1.23 (1.17-1.28)
<2 million won	1.41 (1.35-1.48)		1.40 (1.34-1.47)
<1 million won	1.46 (1.37-1.55)		1.44 (1.36-1.53)
**Area-level income (won)**			
1st quartile: 3.13to 5.58 million		1.00 (Reference)	1.00 (Reference)
2nd quartile: 2.69 to 3.12 million		1.11 (0.91-1.21)	1.11 (1.04-1.19)
3rd quartile: 2.21 to 2.68 million		1.13 (0.90-1.18)	1.14 (1.06-1.24)
4th quartile: 1.32 to 2.21 million		1.07 (0.81-1.06)	1.17 (1.02-1.33)
Regional random variance (SE)			0.062 (0.011)
**Rural**			
**Household income**			
≧3 million won	1.00 (Reference)		1.00 (Reference)
<3 million won	1.17 (1.10-1.25)		1.17 (1.10-1.25)
<2 million won	1.31 (1.23-1.38)		1.31 (1.24-1.39)
<1 million won	1.33 (1.25-1.41)		1.33 (1.25-1.42)
**Area-level income**			
1st quartile: 3.13to 5.58 million		1.00 (Reference)	1.00 (Reference)
2nd quartile: 2.69 to 3.12 million		1.05 (0.92-1.20)	1.06 (0.94-1.20)
3rd quartile: 2.21 to 2.68 million		1.00 (0.89-1.13)	1.07 (0.96-1.19)
4th quartile: 1.32 to 2.21 million		0.85 (0.76-0.95)	0.99 (0.89-1.10)
Regional random variance (SE)			0.033 (0.005)

Data are presented as odds ratio (95% confidence interval).

*Adjusted for individual-level characteristics (age, alcohol intake, BMI, stress perception, depressive mood perception, self-rated health status, family type, marital status).

In men, the lowest area-level income group did not have a significantly lower risk of smoking compared with the highest area-level income group in urban areas (OR, 1.07; 95% CI, 0.81–1.06). However, the lowest area-level income group had a lower risk of smoking than did the highest area-level income group in rural areas (OR, 0.85; 95% CI, 0.76–0.95) (Table 3).

In women, the lowest household income group had a higher risk of smoking than did the highest household income group in both urban and rural areas after adjusting for individual characteristics (urban: OR, 2.38; 95% CI, 2.06–2.76; rural: OR, 1.51; 95% CI, 1.25–1.83). The lowest area-level income group had a lower risk of smoking than did the highest area-level income group in rural areas, but not in urban areas after adjusting for individual characteristics and household income (urban: OR, 0.88; 95% CI, 0.66–1.17; rural: OR, 0.52; 95% CI, 0.39–0.70) (Table 4).

**Table 4:** **Odds ratio for current smoking according to income status in women**

	**Model 1** ^*^	**Model 2**	**Model 3**
**Urban**			
**Household income**			
≧3 million won	1.00 (Reference)		1.00 (Reference)
<3 million won	1.66 (1.44-1.90)		1.66 (1.44-1.91)
<2 million won	2.34 (2.05-2.66)		2.34 (2.06-2.67)
<1 million won	2.37 (2.04-2.74)		2.38 (2.06-2.76)
**Area-level income**			
1st quartile: 3.13to 5.58 million		1.00 (Reference)	1.00 (Reference)
2nd quartile: 2.69 to 3.12 million		1.11 (0.95-1.30)	0.98 (0.86-1.13)
3rd quartile: 2.21 to 2.68 million		1.19 (1.00-1.42)	0.96 (0.82-1.12)
4th quartile: 1.32 to 2.21 million		1.13 (0.83-1.54)	0.88 (0.66-1.17)
Regional random variance (SE)			0.209 (0.004)
**Rural**			
**Household income**			
≧3 million won	1.00 (Reference)		1.00 (Reference)
<3 million won	1.26 (1.03-1.54)		1.29 (1.06-1.58)
<2 million won	1.47 (1.22-1.76)		1.52 (1.26-1.82)
<1 million won	1.45 (1.20-1.75)		1.51 (1.25-1.83)
**Area-level income**			
1st quartile: 3.13to 5.58 million		1.00 (Reference)	1.00 (Reference)
2nd quartile: 2.69 to 3.12 million		0.95 (0.68-1.33)	0.86 (0.62-1.19)
3rd quartile: 2.21 to 2.68 million		0.82 (0.61-1.11)	0.67 (0.50-0.90)
4th quartile: 1.32 to 2.21 million		0.69 (0.52-0.93)	0.52 (0.39-0.70)
Regional random variance (SE)			0.323 (0.069)

Data are presented as odds ratio (95% confidence interval).

*Adjusted for individual-level characteristics (age, alcohol intake, BMI, stress perception, depressive mood perception, self-rated-health status, family type, marital status).

In women, the lowest area-level income group did not have a significantly lower risk of smoking than did the highest area-level income group (OR, 1.13; 95% CI, 0.83–1.54). The lowest area-level income group had a lower risk of smoking than did the highest area-level income group in rural areas (OR, 0.69; 95% CI, 0.52–0.93) (Table 4).

## Discussion

The results show that there are differences in smoking between area-level income groups. In men, the lowest area-level income group had higher age-adjusted prevalence of smoking in urban areas, however, in women, the highest area-level income group had higher age-adjusted prevalence of smoking in rural areas. In men, the lowest area-level income group had a higher risk for smoking in urban areas. In women, the lowest area-level income group had a lower risk for smoking in rural areas.

The results show strong independent effects of household income on smoking in both men and women. The lowest household income group had a higher risk of smoking than did highest household income group in both urban and rural areas. This result is similar to findings of many previous studies showing that the prevalence of smoking in low SES groups is higher than that in high-SES groups [23,24]. Some previous studies have reported that low-SES groups are more likely to try smoking and become regular smokers, and less likely to quit smoking [25,26]. Some explanations have been proposed for this phenomenon. First, among low-SES people, smoking may serve as a coping mechanism that helps them to deal with the difficult and stressful aspects of their daily lives [12,27]. Second, unfavorable material conditions may promote smoking among low-SES people [28,29]. Surviving economic disadvantages can place excessive strain on an individual [30], and material conditions limit the possibilities engaging in healthy behaviors [31]. Third, people living in low-SES circumstances may lack the knowledge, information and resources for healthy behavior. A previous study reported that education that improves health-related knowledge influences individuals to adopt a healthier lifestyle [32]. People with low SES may be less knowledgeable about the harmful effects of smoking [33]. Finally, smoking cessation was low in low-SES smokers. Lower SES is associated with lower social support and integration, and this might reduce the chances of successfully quitting smoking [34,35]. Low-SES smokers appear to be more concerned with current than with future health issues, and they are less likely to intend to quit or to have a sense of duty to quit smoking [36,37].

In this study, the risk of smoking decreased according to increasing individual-level and area-level income status in urban men. Some previous studies have reported that the prevalence of smoking was higher in both men and women in socioeconomically disadvantaged areas [38]. Individuals living in socioeconomically disadvantaged areas have been found to be more likely smoke, perhaps because daily life in a lower-SES group results in greater exposure to unfavorable circumstances [39]. A previous study suggested that the neighborhood influences health, and the mechanisms of influence include availability and accessibility of health service, infrastructure deprivation, prevailing attitudes towards health and health-related behaviors, stress, and a lack of social support [40]. Living with low SES may be a stressful situation, and smoking may serve as a coping response to that situation. A previous study suggested that the same levels of stress might have different effects on different socioeconomic groups because of different perceptions and coping strategies, and that smoking is one response to stress induced by unfavorable socioeconomic circumstances [41]. Area of low SES might be the areas with a lack of social support. One study reported that smoking in areas with low social participation is higher than that in areas with high social participation [42].

Neighborhood effects may be heterogeneous across different individual SES [43]. Harmful effects of low individual SES could be intensified for those who live in poor neighborhood [44]. On the other hand, poor people living in affluent neighborhoods may be stressed from perceived income inequality and thus suffer from relative deprivation and relative standing [44,45]. However, in this study, area-level income did not affect differently the smoking behaviour of people with high and low individual-level income. One previous study has reported that persons of low individual-level income were not more vulnerable to area affects on smoking those of high individual-level income [46]. Few previous studies have investigated interactions between area-level and individual-level characteristics, and they have found no consistent evidence of interactions [47,48].

Previous studies have found an association between smoking and area of residence [49-51] and have reported area effects independent of individual-level socioeconomic effects on smoking. The present study also showed such effects. Individual smoking was associated with area-level income status after adjusting for household income and individual characteristics. However, these effects differed according to sex and region. The independent effects of area-level income status on smoking were significant in urban areas in men and in rural areas in women, and these associations were in the opposite directions in men and women.

If household income only affected individual smoking, then smoking would be more common in areas with lower area-level income. However, in the present study, the risk of smoking in women increased with increasing area-level income in rural areas. Although it was not statistically significant, women’s risk of smoking also showed a tendency to increase according to increasing area-level income in urban areas.

One possible explanation is that traditional gender roles and norms in women are rapidly changing in Korea. Norms for smoking behavior in women are changing, and these changes are especially likely to occur easily in wealthier, more educated, and developed areas. Although traditional gender roles result in social pressure against women’s smoking [52], if smoking is socially acceptable and more prevalent among women in an area, women living in this area are more likely to adopt the same lifestyle. One study found that the risk of smoking in women increased in areas of high social class, suggesting that the cultural restrictions on women’s smoking that remain in areas of low social class may cause decreased smoking [13].

Another explanation is that patterns of women’s smoking behaviors in urban are similar to smoking behaviors in men, so the effects of area-level income on smoking were attenuated. One previous study proposed four distinct stages in smoking epidemic model [53]. According to this model, smoking epidemic in Korea seems to be third stage that men prevalence begins to decline but women prevalence continues to rise constantly until end of the stage.

### Strengths and limitations

This study had several strengths. First, it was population-based and included a relatively large sample size. The subjects of this study were representative, as we used complex, multistage, probability sampling design. Second, a multilevel regression model was used to evaluate the associations of individual-level and area-level income status with individual smoking. Compared with single-level analysis, multilevel analysis has the advantage of being able to reduce statistical problems including aggregation bias, estimation error for standard error, and heterogeneity of regression [54]. Third, this analysis was performed separately by sex and region to evaluate different effects of area-level income status on smoking. Associations between health and related behaviors and geographical area can be different for men and women [55].

The association between income status and smoking indicates that income status may be an important risk factor for smoking. However, some limitations should be mentioned. First, this cross-sectional study can only suggest causal relationships. Second, information on income and smoking status was self-reported, and area income was calculated as average income of area living individual. But, there is little available area income information at the district level in Korea. Third, although many confounding factors were adjusted, unrecognized confounding factors may have affected to both income and smoking. Fourth, associations between smoking and income status were evaluated, but income status is only one socioeconomic indicator. The socioeconomic position of individuals and areas are multidimensional, and associations between socioeconomic position and health behavior vary. This may mean that other area characteristics have contextual effects on smoking, and other dimensions of socioeconomic indicators may need to be evaluated. Fifth, smoking-related area factors including availability of cigarettes and anti-smoking policies were not evaluated. Finally, geographic and cultural differences between urban and rural areas may have different effects on the association between income status and smoking.

## Conclusions

Household income was associated with smoking, and area-level income status was associated with smoking independent of household income status. Effects of area-level income on smoking differed by sex and region. In men, the lowest area-level income group had a higher risk of smoking in urban, but not in rural areas. However, in women, the lowest area-level income group had a lower risk of smoking in rural areas, but not in urban areas. Findings of this study suggest that area-level income may influence individual smoking along with household income status. Future studies to identify effects of other area-level characteristics on smoking are needed to determine the mechanisms of area effects on health behavior at the individual-level.
